# Attitudes towards women in the military and their relation to both quantity and quality contact with female leaders

**DOI:** 10.3389/fpsyg.2024.1282835

**Published:** 2024-03-13

**Authors:** Adelheid A. M. Nicol, Amélie Mayrand Nicol

**Affiliations:** ^1^Military Psychology and Leadership Department, Royal Military College of Canada, Kingston, ON, Canada; ^2^Departments of Biology and Neuroscience, University of Carleton, Ottawa, ON, Canada

**Keywords:** contact quality, contact quantity, intergroup anxiety, perspective-taking, empathy, attitudes toward women in the military, gender roles

## Abstract

Contact experiences with women in senior leadership roles are important for creating acceptance of women in organizations dominated by men, such as the military, as leadership roles are considered demanding, requiring numerous agentic qualities that are often ascribed to men. The military lacks women in leadership levels within its organization. We wished to determine whether quality and quantity contact with women in leadership positions reduces intergroup anxiety, increases empathy and perspective-taking, and subsequently creates more favorable attitudes toward women in the military. This was examined in three studies, one with a military sample consisting of men (*n* = 95), another with a civilian sample of men (*n* = 367), and a third study with a civilian sample of women (*n* = 374). Our findings revealed that quality contact was related to attitudes toward women in the military for all three samples. Results from the indirect effects tests conducted for the civilian male and female samples revealed that for civilian men, intergroup anxiety demonstrated a significant indirect effect between quantity contact and attitudes toward women in the military, while both intergroup anxiety and perspective-taking demonstrated significant indirect effects between quality contact and attitudes toward women in the military. Furthermore, both quantity and quality contact demonstrated significant direct effects. On the other hand, results revealed that for civilian women the only significant relation was the direct effect between quality contact and attitudes toward women in the military. Intergroup anxiety, perspective-taking, and empathy did not demonstrate any indirect effects for the civilian women sample. Thus, given that interactions with women in leadership positions are related to views of women in the military, research should further explore the role of contact for women in non-traditional work roles.

## 1 Introduction

Because women have traditionally occupied gender roles related to domestic work and care-giving, they are often not viewed as leaders (Eagly and Karau, 2002; Eagly and Carli, 2007; Smith et al., 2019). This is despite the fact that they have demonstrated similar or better performance as leaders (Boldry et al., 2001; Eagly and Carli, 2003; Paustian-Underdahl et al., 2014; Chadwick and Dawson, 2018; Farh et al., 2020). Men are frequently viewed more favorably in leadership roles (Kiser, 2015) as masculine stereotypes are ascribed to them and deemed more suitable (Kossek and Buzzanell, 2018; Samo et al., 2019; Smith et al., 2019; Friedmann and Efrat-Treister, 2023). This often leads to women being disadvantaged in leadership roles in male-dominated organizations (Eagly et al., 1995; Heilman, 1995; Eagly and Carli, 2007; Offermann and Foley, 2020; Wells et al., 2020) like the military (Vogt et al., 2007; Young and Nauta, 2013).

Leadership in the military is extolled and values and performance criteria are shaped by stereotypical definitions of maleness (Durning, 1978; Deng et al., 2023a,b). It is an environment that values dominance, power, being tough, physical fitness, competition and aggression (Vogt et al., 2007; Nicol et al., 2011). Therefore, the military attracts more individuals with traditional attitudes and values (Vogt et al., 2007). The military nurtures hierarchical views (Nicol et al., 2007; Van Wijk, 2011), and tends to be more sexist as women are viewed as inferior members who need to be protected (Young and Nauta, 2013). In traditionally male-dominated environments, such as the military, women are more likely to be sexually harassed, to have mostly male superiors, and to occupy traditional gender roles in medicine or administration (Vogt et al., 2007; Office of the Auditor General of Canada, 2016; Daphna-Tekoah et al., 2021; Arbour, 2022). Women are still considered by many as unsuitable for the military, particularly in leadership roles (e.g., Torres-Reyna and Shapiro, 2002; Young and Nauta, 2013). They are viewed as obstacles to effectiveness who lack agentic qualities and physicality in order to get the job done (Deng et al., 2023a,b). These views fuel the existing masculine, patriarchal norms that define the military as masculine (Deng et al., 2023a,b).

Perceptions of women being unsuitable for the military may explain the low proportion of women in the military. Presently, in Canada, 16.1% of the Canadian Armed Forces are women (Government of Canada, 2023). This is higher than the NATO average rate of 13% (NATO, 2020). Many other countries see low rates, as well. For instance, in the United States, 17.5% of active duty members were women in 2022 (Department of Defense, 2022). In 2021–2022, women represented 20.1% of Australian Defence Force members, with 16.6% in senior ranks (Australian Government Defence, 2022). In South Korea, 1.6% of active duty personnel were women in 2015, this was expected to rise to 5.6% in 2020 (Obradovic, 2015). In Sweden, women represent 7% of the professional military officers (Persson and Sundevall, 2019). In Israel, female officers represent only 14% of high-ranking officers yet form nearly 34% of the Israel Defense Forces. Although women successfully occupy many different positions, such as combat arms in numerous countries (e.g., Hardt and von Hlatky, 2020; Loukou, 2020; Moore, 2020; Tharion et al., 2023), their low numbers are problematic. Increasing the number of women in the military is important to foster necessary culture change, change attitudes regarding women’s suitability in military roles, provide role models to other women, and to adapt to society’s call for greater equity, diversity, and inclusivity in the workplace (Soules, 2020; Deng et al., 2023b).

Negative attitudes toward women in the military may be partly due to the lack of female representation in leadership roles. Contact theory suggests that greater contact with outgroup members improves attitudes toward those members (Allport, 1954). This theory was used to explain the improvement in attitudes toward women after their integration in the U.S. military (Durning, 1978; Stevens and Gardner, 1987). Research exploring the role of contact with women and its relation to attitudes is scant; none have examined whether exposure to women in leadership roles improves attitudes toward women in the military. The purpose of this research was to examine whether contact with women in leadership roles was related to attitudes toward women working in the armed forces, as well as to assess the indirect effects of intergroup anxiety, empathy, and perspective-taking in explaining that relation.

### 1.1 Contact theory and attitudes toward women in the military

Individuals with little experience interacting with ‘outgroup’ members typically express more negative attitudes toward them. Allport’s (1954) original formulation of contact theory suggested that contact with outgroup members (typically individuals of minority group status) can lead to more favorable views of those members and reduce intergroup prejudice. Specifically, four conditions are important for intergroup contact to influence prejudice: there needs to be equal status between the members of the ‘ingroup’ and the ‘outgroup’; both groups should pursue a common goal; cooperation is required; and the contact is supported by the existing culture, law, and/or relevant authority figure(s) (Pettigrew and Tropp, 2006). Pettigrew and Tropp’s (2006) meta-analysis of contact research supported the basic premise of Allport’s contact theory and found that contact improved intergroup attitudes. The four conditions resulted in an optimal attitude change toward outgroup members, but those conditions were not required in order for prejudice reduction to occur (Pettigrew and Tropp, 2006). Since Pettigrew and Tropp’s meta-analysis, extensive research with different samples, different target outgroups, and different measures of attitudes have demonstrated the effectiveness of contact theory (Smith et al., 2009; Lemmer and Wagner, 2015; Banas et al., 2020).

Very few studies examined whether contact plays a role in attitudes toward women (e.g., Durning, 1978; Cheatham, 1984; Ivarsson et al., 2005; Taschler and West, 2017; Jenkins et al., 2023). This is probably because women and men interact constantly in personal and work life and have many cross-gender relationships (Taschler and West, 2017). Thus, women are not generally viewed as ‘outgroup’ members as they form equal numbers with men, in general, within society (Taschler and West, 2017). However, because women in the military, or in leadership roles, form a minority group, we propose that contact theory can help explain attitudes toward women in the military. Banas et al. (2020) suggest that positive contact results in a re-evaluation of the whole group. Contact increases inclusion of outgroup members in the in-group, fosters critical assessment of the ingroup’s attitudes and behaviors, and decreases perceptions that the outgroup poses a threat (Cakal et al., 2021). Thus, increased contact with women in leadership positions, where women are responsible for making independent decisions, and have the responsibility of others and their organization on their shoulders, may result in viewing women as more suitable to the military.

Research at various academies provides some support for the relevance of contact theory to explain attitudes toward women. Durning (1978) examined the influence of integrating women in the military. Durning (1978) found that after five months of serving in platoons, the men in mixed-sex platoons had more egalitarian attitudes; however, attitudes toward women were not improved. Cheatham (1984), studied female integration at the U.S. Coast Guard Academy. Cheatham (1984) found that attitudes toward women in the military improved after exposure to more women. One of the first studies that directly measured the relation between contact with women, by including a measure of contact, was conducted by Ivarsson et al. (2005). They examined attitudes toward women in the military in male officers of the Swedish Armed Forces and included contact as a potential predictor. Contact was measured by asking participants how many female military personnel they knew, how often they interacted with them, how long they interacted with them, and the quality of their experience. Using regression analyses, Ivarsson et al. (2005) found that education, rank, sexism, quantity of contact, as well as quality contact were the best predictors of attitudes toward women in the military (frequency and length of contact did not predict over and above these variables). Thus, although explanatory mechanisms were not studied, contact with women in the military appears to be important to take into consideration when considering attitudes toward women in the military (Ivarsson et al., 2005).

The research examining the influence of contact with women in positions of authority and its relation to attitudes toward women is scant. Taschler and West (2017) found that contact with female leaders resulted in less sexism and a reduction in rape myth acceptance. Delgado-Iglesias et al. (2019) found, for a sample of female employees, that experience with working with a female boss predicted attitudes toward female leaders. Experience with working with a female boss and attitudes toward female leaders were unrelated in the male sample, however the use of a single, dichotomous variable to assess contact with female leaders may explain the nonsignificant findings for the male sample. Baldner et al. (2022) found that quality contact with female managers predicted attitudes toward female managers, but that quantity contact did not in a combined sample of men and women (differences between men and women were not examined). More research is required to determine whether there is a relation between contact with female leaders and attitudes toward women in the military, as well as study intermediary variables that may explain this relation.

### 1.2 Mediators of contact

Contact is a distal predictor of attitudes, as its relation with attitudes can be explained by several mediators (Aberson and Haag, 2007). Pettigrew and Tropp (2008) summarized the research that examined mediators of the contact-attitude relation (note, Pettigrew and Tropp did not distinguish between different kinds of contact). They found that contact reduced intergroup anxiety, increased empathy/perspective-taking toward the stigmatized group, and increased knowledge about the members of the stigmatized group. All three mediators (i.e., intergroup anxiety, empathy/perspective-taking, and knowledge) were subsequently related to more favorable attitudes toward the outgroup and were important for explaining the contact-prejudice relation (e.g., Turner et al., 2007; Pagotto et al., 2010; Swart et al., 2010, 2011; Vezzali and Giovannini, 2012; Pagotto and Voci, 2013; Mak et al., 2014; Cakal et al., 2021). Intergroup anxiety and empathy/perspective-taking were identified as the strongest mediators (Pettigrew and Tropp, 2008). Thus, our research focussed on these stronger mediators: intergroup anxiety, empathy, and perspective-taking.

Intergroup anxiety is an experience of negative emotions when interacting with, or even imagining interactions with, outgroup members (Stephan, 2014). It is predictive of attitudes toward outgroup members, behavioral intentions, and intercultural communication (e.g., Riek et al., 2006; Stephan, 2014; Logan et al., 2016; Chen and Zhang, 2022; Kanamori et al., 2022; Zhang et al., 2023). Initially contact may increase intergroup anxiety, but with enough contact experiences, it will reduce it (Aberson and Haag, 2007). Individuals not having a lot of experience with women in leadership roles may find the prospect of interacting with women in the work environment anxiety-provoking. They may feel unsure as to how to act around the outgroup or what to expect from the interaction. We expect intergroup anxiety to function as an important mediator in this research.

We wished to examine the unique contributions of perspective-taking and empathy. Thus, we measured them separately. Perspective-taking is very important for social relations, leads to altruistic behaviors, decreased aggression toward others, decreased egocentrism, and increased situational attributions for others (Davis et al., 1996). Perspective-taking is considered a cognitive ability, whereby an individual takes another person’s view of things to understand what they are going through (Matera et al., 2021; Bobba and Crocetti, 2022). Perspective-takers will use information regarding the outgroup to adjust their own behavior accordingly (Galinsky et al., 2008) and to focus on the development and maintenance of social relations with others (Wang et al., 2014). Those scoring high on perspective-taking may be better able to merge schemas of themselves with schemas of others (Galinsky et al., 2008). Research has demonstrated perspective-taking to be an important predictor of attitudes, emotions, and behavioral intentions toward outgroup members (e.g., Shih et al., 2009; Persson and Hostler, 2021). It is also an important mediator between contact and attitudes or behavior toward outgroup members (e.g., Vezzali and Giovannini, 2012; Cakal et al., 2021).

Empathic concern refers to orienting toward others, along with experiencing emotions that match or reflect feelings of others (Bobba and Crocetti, 2022). Empathy permits an individual to connect emotionally with others (Matera et al., 2021). How much empathy we feel and which groups we feel empathy toward are influenced by many factors. For instance, how much exposure we have to outgroups, directly and indirectly through cross-friendships or the media, can influence feelings of empathy (Banas et al., 2020). Research has demonstrated empathy to be an important predictor of attitudes, emotions, and behavioral intentions toward outgroup members (e.g., Pedersen et al., 2004; Pagotto et al., 2010; Pagotto and Voci, 2013), as well as an important mediator between contact and attitudes (Swart et al., 2010, 2011).

Many studies examining perspective-taking and empathy either combined the constructs and did not provide results for their independent effects (e.g., Bagci et al., 2019), or studied one or the other but not both (e.g., Vezzali et al., 2010; Vezzali and Giovannini, 2012; Visintin et al., 2017). When both perspective-taking and empathy were measured, varying results were obtained. Gloor and Puhl (2016) found that when both empathy and perspective-taking were experimentally manipulated there were improvements in empathy and affective reactions regarding the outgroup. Wang et al. (2014) found empathy was not strongly related to willingness to engage in contact while perspective-taking was. Other research found perspective-taking and empathy performed differently with variables not assessing prejudice (Chartrand and Bargh, 1999; Galinsky et al., 2008). Chartrand and Bargh (1999) found that those scoring high on perspective-taking engaged in more behavioral mimicry than did those scoring low on perspective-taking while there were no differences for those high or low on empathic concern. Galinsky et al. (2008) found perspective-taking to be useful for negotiations, while empathy was not. Different neural networks, different correlates, and different developmental paths describe empathic concern and perspective-taking (Bobba and Crocetti, 2022). Thus, the inclusion of both perspective-taking and empathy in order to study their unique role in the contact-attitudes toward women in the military relation is important in order to understand the underlying processes involved.

### 1.3 Quantity and quality of contact

There is extensive research demonstrating contact’s effectiveness in reducing prejudice, but a lack of research examining its role in attitudes toward women. Therefore, we wished to determine whether both quantity and quality contact with women in positions of power or authority reduces intergroup anxiety, increases perspective-taking, and increases empathy, which subsequently creates more favorable attitudes toward women in the military.

Quantity of contact refers to the number of interactions with outgroup members and/or the number identified as friends (Binder et al., 2009; Kanamori et al., 2022). Quality contact refers to the extent to which experiences with the outgroup members are positive (Kanamori et al., 2022). Pettigrew and Tropp (2008) did not distinguish between these two kinds of contact in their meta-analyses. Some researchers have studied either quality or quantity contact (e.g., Pagotto et al., 2010; Pagotto and Voci, 2013; Cakal et al., 2021), or combined these two forms of contact into a single measure of contact (e.g., Vezzali and Giovannini, 2012). Research suggests that findings may differ, depending upon the type of contact measured.

When measured separately, research found that quality and quantity contact contribute differentially to describing attitudes, social distance, and intergroup anxiety (e.g., Voci and Hewstone, 2003; Vezzali and Capozza, 2011; Méndez Fernández et al., 2022). For example, quantity may be better related with implicit attitudes while quality is more strongly related to explicit attitudes (e.g., Tam et al., 2006; Prestwich et al., 2008; Kanamori et al., 2022). In some instances, quality of contact was more effective at decreasing prejudice than quantity of contact (e.g., Plant and Devine, 2003; Turoy-Smith et al., 2013; Mak et al., 2014; McKeown and Psaltis, 2017; Zhang et al., 2023). This may be because quantity contact could be influenced by the quality of the contact and whether there is perceived support for the contact (Zhang et al., 2023). Thus, incorporating both measures will provide a more nuanced understanding of the role of contact in our research.

### 1.4 Current research

**Figure 1:**
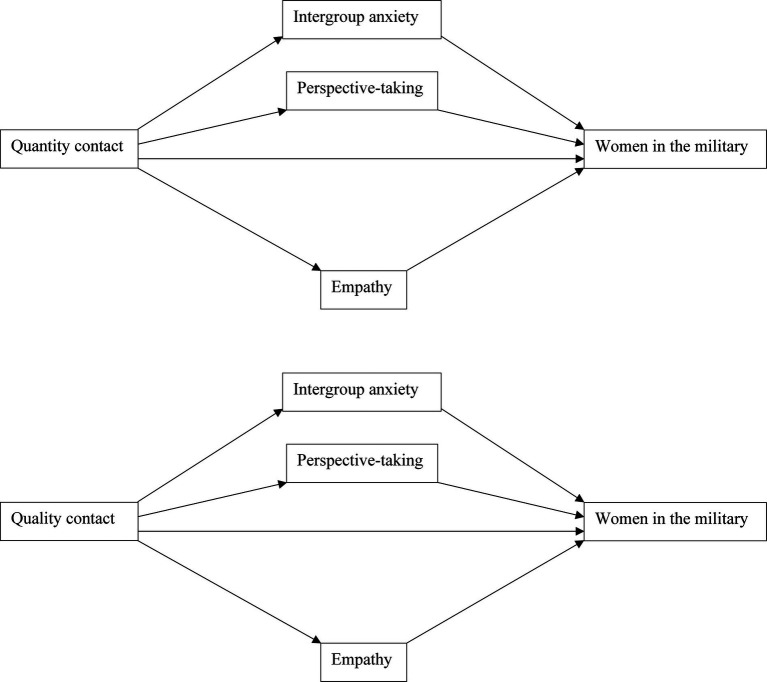
The indirect effects models.

Given the lack of research examining the role of contact with women in influencing attitudes toward women, we undertook three studies to examine the relation between these two variables. Specifically, we studied the relation of contact with women in leadership positions (both contact quality and quantity) with attitudes toward women in the military. We further examined the indirect effects of intergroup anxiety, perspective-taking, and empathy on those relations (See Figure 1). This would determine whether they function in a mediating role. The first study examined these relations in a Canadian military male sample. The second study examined these relations in a Canadian civilian male sample and the third study examined these relations in a Canadian civilian female sample. Studying the relations separately for men and women, as well as including a military sample, provides information as to whether there are unique mechanisms at play for these samples.

## 2 Study 1

Young and Nauta (2013) suggested that military men may have more traditional views than civilians as their opportunities to work with women leaders are limited. Indeed, Young and Nauta (2013) found that military-affiliated students are less approving of women in combat than civilian students; however, they found no differences in attitudes toward women in the military. Matthews et al. (2009) found military cadets had more unfavorable attitudes toward women in certain military occupations than did civilians. Although the purpose of our research was not to study differences between civilian and military samples, Matthews et al’s. (2009) and Young and Nauta’s (2013) findings help understand the context for this sample and suggest the importance of studying the role of contact with female leaders in a male, military sample. Young, male cadets represent a portion of the officers of the future who will help guide and propel culture change. We expected both quantity and quality of contact to be significantly related to attitudes toward women in the military, and all three mediators to play an intermediary role in predicting attitudes toward women in the military.

*H1:* Quantity contact would predict attitudes toward women in the military. Intergroup anxiety, perspective-taking, and empathy would demonstrate significant indirect effects.

*H2:* Quality contact would predict attitudes toward women in the military. Intergroup anxiety, perspective-taking, and empathy would demonstrate significant indirect effects.

### 2.1 Method

#### 2.1.1 Participants and procedure

This study received approval from the Research Ethics Board of the Royal Military College (RMC) of Canada. The participants, military cadets registered full-time in the Regular Officer Training Plan (ROTP), were recruited through internal RMC email. Only self-identified men were asked to voluntarily participate because of the small population of cadets at this institution and the small proportion of women who attend. RMC is the largest military educational college in Canada, with approximately 1,200 cadets, 78% men and 22% women. Officer and naval cadets attend the college in order to complete an undergraduate degree. Upon successful graduation, cadets become commissioned as officers in the Canadian Armed Forces.

The items from measures were presented in random order on the Survey Monkey platform. The demographic questionnaire was presented last and attention check items, modeled after Kam and Meyer (2015) work, were presented throughout.

A total of 208 participants clicked on the survey study link but only 95 participants’ data were complete. Three participants did not wish to consent to participate, 53 consented to the survey but then did not answer any questions, 43 indicated that they wished to participate but did not answer most of the questions, 12 did not self-identify as a man or did not indicate their gender, and 2 failed one or more attention check items. Seven participants had one or two items missing and these were replaced by the mean score for that scale. The participants’ ages ranged between 17 and 26 (*M* = 20.0, *SD* = 1.90). Of these respondents, 33.7% were first years, 20.0% were second years, 21.1% were third years, 16.8% were fourth years, and 8.4% were fifth years. Furthermore, 83.2% indicated English was their first language while 16.8% indicated that French was their first language.

#### 2.1.2 Measures

##### 2.1.2.1 Quantity contact

Taschler and West’s (2017) five quantity contact items were employed, each using a 1 to 5 scale (response options varied for each item). The instructions were adapted to read, “We are interested in your **experiences with women in positions of power or authority**, or women who are more senior than you at the college.” A sample item is, “Right now, how many of your close friends are women like these?” All five items were added to create a total score; a high score reflects greater contact. Cronbach’s alpha was 0.73 for this scale. The range for contact quantity scores (total scores) in this sample was 6 to 21.

##### 2.1.2.2 Quality contact

Taschler and West’s (2017) six items assessing the quality of contact with women in positions of power or authority were employed, each using a 1 (*strongly disagree*) to 7 (*strongly agree*) scale. As with the quantity contact measure, the instructions were adapted to read, “We are interested in your **experiences with women in positions of power or authority**, or women who are more senior than you at the college.” A sample item is, “In general, my experiences with these women have been pleasant.” All six items were totalled; a higher score reflects greater quality contact. Cronbach’s alpha was 0.86 for this scale. The range for contact quality (total scores) in this sample was 11–42.

##### 2.1.2.3 Intergroup anxiety

Stephan and Stephan’s (1985) intergroup anxiety scale was adapted for this study to measure anxiety with working with women. The instructions were modified to read: “If you were the only man interacting with women (e.g., talking with them, working on a project with them), how would you feel compared to occasions when you are interacting with only men?” This measure consists of 11 items, and respondents are asked to indicate the extent to which they feel less (1) or more (10) of each feeling. A total score reflects greater anxiety in interacting with women. Cronbach alpha was 0.80.

##### 2.1.2.4 Perspective-taking

Perspective-taking was measured using the nine-item measure by Davis (1980). A sample item is, “I believe that there are two sides to every question and try to look at them both.” Participants responded to the items on a Likert scale of 1 (*strongly disagree*) to 7 (*strongly agree*). Cronbach’s alpha was 0.76. Perspective-taking scores were calculated as the total across all items, after reverse-coding three items. A high score indicates that the participant is high in levels of perspective-taking.

##### 2.1.2.5 Empathy

Empathy was measured using the 14-item measure by Davis (1980), of which four are reverse-coded. A sample item is, “I am often quite touched by things that I see happen.” Participants responded to the items on a Likert scale of 1 (*strongly disagree*) to 7 (*strongly agree*). Cronbach’s alpha was 0.74. Empathy scores were calculated as the total across all items, after reverse-coding the four items. A high score indicates that the participant is high in levels of empathy.

##### 2.1.2.6 Attitudes toward women in the military

Eleven items were taken from the 12-item Women in the Military scale by Hurrell and Lukens (1995) to assess attitudes toward women in the military. This measure has three factors: ability to perform in comparison with men, women having children while in the military, and family role and combat (Hurrell and Lukens, 1995). This study employed a total score across all three factors. Individuals responded on a 1 (*strongly disagree*) to 7 (*strongly agree*) scale. An example item is, “Women have as much to offer in the military service of their country as men.” The following item from the original scale was not included, “If reinstated, both men and women should be subject to the draft.” Six items were reverse-coded. A high score indicates a favorable attitude toward women in the military. Cronbach’s alpha was 0.83.

##### 2.1.2.7 Attention check

One attention-check item from Kam and Meyer (2015) work was included in each scale of the survey except for the quantity contact and intergroup anxiety scales. A sample item is: “This is to make sure there is no random responding, please select ‘strongly disagree’ on the following scale.” Participants who incorrectly answered one or more of these items were removed from subsequent analyses.

##### 2.1.2.8 Demographics information

Participants were asked to answer demographic questions regarding their age, year at RMC, gender, enrollment plan (to ensure they were, indeed, undergraduate students), and first official language.

### 2.2 Results and discussion

All analyses were conducted using the SPSS Version 28 statistic software. Normality and linearity assumptions were met and skewness (−2 to +2) and kurtosis (−7 to +7) were within the acceptable ranges. One-tailed Pearson correlations were calculated between the study variables (see Table 1). Correlations were based on 1,000 bootstrap samples.

**Table 1:** Correlation between study variables for male military cadets.

Variable	QnC	QlC	IA	PT	E	ATWITM	A	YATC
Quantity contact	0.73							
Quality contact	0.10	0.86						
Intergroup anxiety	−0.26**	−0.21*	0.80					
Perspective-taking	0.11	0.04	0.02	0.76				
Empathy	0.14	0.24**	−0.19*	0.41***	0.74			
Attitudes towards women in the military	0.05	0.20*	−0.13	0.04	0.25**	0.83		
Age	0.13	−0.07	−0.03	0.29**	0.11	0.05	--	
Years at the college	0.25**	−0.09	0.06	0.34***	0.13	0.08	0.71***	--
Mean	2.28	5.39	6.15	4.72	4.83	5.58	20.00	2.46
SD	0.63	1.01	1.01	0.79	0.65	0.93	1.91	1.34

QnC, quantity contact; QlC, quality contact; IA, intergroup anxiety; PT, perspective-taking; E, empathy; ATWITM, attitudes towards women in the military; A, age; YATC, years at the college. Cronbach’s alpha for measures on the diagonal.

*p < 0.05; **p < 0.01; ***p < 0.001.

Although the sample size was too small to conduct a test of the mediation models for quantity contact, quality contact, and attitudes toward women in the military, the significant relations found between quality contact and attitudes toward women in the military were in the hypothesized directions. Quality of contact demonstrated stronger relations with more variables than did quantity of contact. Quality of contact was found to be significantly related to intergroup anxiety, empathy, and attitudes toward women in the military, while quantity contact was related only to intergroup anxiety. This supports research that has found quality contact to be particularly relevant to explicit attitudes (e.g., Tam et al., 2006; Prestwich et al., 2008; Kanamori et al., 2022; Zhang et al., 2023).

Research has shown intergroup anxiety and empathy/perspective-taking to be important explanatory factors as they act as mediators between contact and attitudes toward outgroup members (Pettigrew and Tropp, 2008). We found significant relations between empathy and quality contact as well as empathy and attitudes toward women in the military, providing preliminary support for empathy’s important role in reducing negative attitudes toward women in the military.

Perspective-taking was not significantly related with our contact variables or our mediators, suggesting that it may not play a role for young, military men. We do not know why different results were found for empathy and perspective-taking. Research has shown the importance of development for both empathy and perspective-taking (e.g., O’Brien et al., 2013; Van der Graaff et al., 2014). It is possible that development may explain these results given that only young male adults were studied. These findings suggest the importance of studying empathy and perspective-taking separately.

This study provides preliminary support for the application of contact theory to attitudes toward women in the military. Our findings suggest the importance of ensuring that young, male military cadets gain quality experiences with women in leadership roles. Even though women represent approximately 22% of the cadet population at RMC (Scoppio et al., 2020), which is higher than the proportion of women in the Canadian Armed Forces (16.3%; Government of Canada, 2022), it would seem that interacting with women in positions of authority has an important role in determining cadets’ views of women’s suitability for the military. This might be because intergroup anxiety is reduced and empathy is increased.

## 3 Study 2

It was important to examine the hypothesized relations in a larger sample in order to test the mediation models. Thus, we sampled a larger, civilian population of men. The military interacts frequently with civilians, and civilians’ attitudes toward women in the military help form the underlying cultural framework by which to understand those interactions as the norms and ideologies interact (Shields, 2020). Indeed, civilians’ attitudes toward women in the military can inform policies and programs delivered to increase representation (e.g., Harel-Shalev, 2021). As in Study 1, we tested whether quality and quantity contact with women in positions of authority were related to attitudes toward women in the military and whether intergroup anxiety, empathy, and perspective-taking functioned as intermediaries.

*H3:* Quantity contact would predict attitudes toward women in the military. Intergroup anxiety, perspective-taking, and empathy would demonstrate significant indirect effects.

*H4:* Quality contact would predict attitudes toward women in the military. Intergroup anxiety, perspective-taking, and empathy would demonstrate significant indirect effects.

### 3.1 Method

#### 3.1.1 Participants and procedure

This study received approval from the RMC Research Ethics Board. The participants were recruited through Prolific, a crowdsourcing service where participants were paid for engaging in research (maximum of £1.50). Only self-identified Canadian men were asked to voluntarily participate. Canadian participants were sought to control for possible differences due to culture. Access to the study was via Survey Monkey where items were presented randomly to all participants. The demographic questionnaire was presented last and attention check items of Kam and Meyer (2015) were presented throughout.

A total of 410 participants clicked on the survey study link but only 367 participants’ data were complete. One participant did not wish to consent to participate, 10 consented to the survey but then did not answer any questions, 13 identified as having military experience, 19 failed one or more attention check items. The participants’ ages ranged between 18 to 76 (*M* = 35.7, *SD* = 11.87, median of 34) with 0.5% not having a high school education, 21.3% having completed high school as their highest level of education, 14.2% having completed a certificate, 49.2% having a bachelor’s degree, and 14.7% with a post undergraduate education. The average number of years of work experience was 14.65 (SD = 11.01) with a median of 13 years, 91.3% declared English as their first language, 4.2% declared French, and 4.5% declared other. The range for contact quantity scores (total scores) in this sample was 5–21, while contact quality (total scores) was 11–42.

#### 3.1.2 Measures

All measures were identical to those described in Study 1, except the instructions for both the quantity and quality contact measures read, “We are interested in your experiences with women in positions of power or authority, or women who are more senior than you occupationally.” Furthermore, in addition to asking for age and language as demographic information, participants were also asked their highest level of education, the number of years of work experience that they had, and whether they had work experience in the Canadian Armed Forces or other military.

### 3.2 Results and discussion

**Figure 2:**
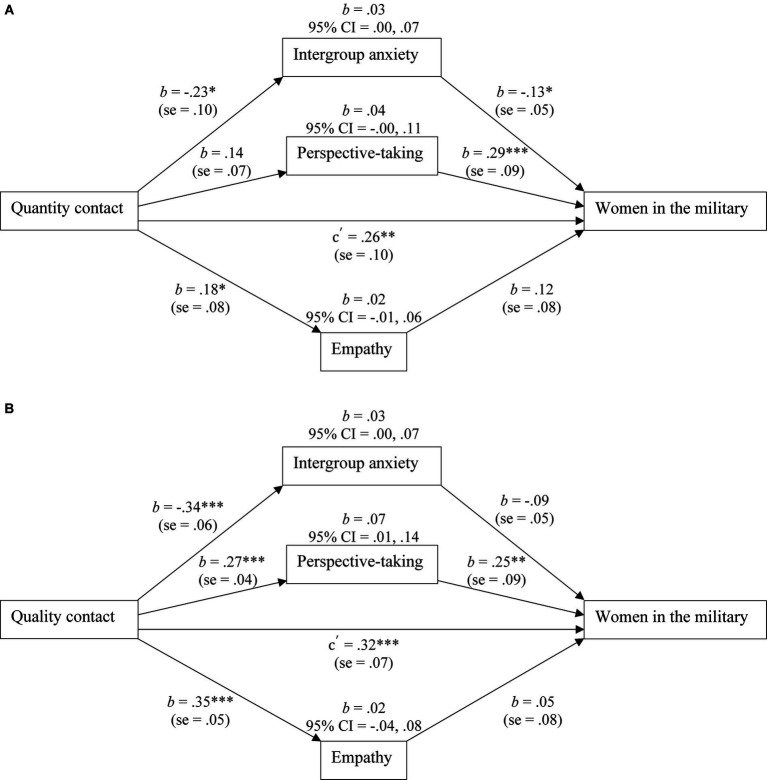
Men civilian sample: the indirect effect test of intergroup anxiety, perspective-taking, and empathy on the association between quantity contact **(A)** or quality contact **(B)** and attitudes towards women in the military. All path values represent unstandardized regression weights. CI = confidence interval. *p < 0.05; **p < 0.01; ***p < 0.001.

One-tailed Pearson correlations were calculated between the study variables (see Table 2). Correlations were based on 1,000 bootstrap samples. Significant intercorrelations were found for all study variables. We analyzed the parallel indirect effects of intergroup anxiety, perspective-taking, and empathy to determine whether they account for unique variance in the association between contact quantity and attitudes toward women in the military or between contact quality and attitudes toward women in the military (see Figure 2 for full model results and path values). The two indirect effects models were tested using model 4 of the PROCESS macro v4.0 (Hayes, 2022). These models were run with 95% confidence intervals and 5,000 sample bootstrapping.

**Table 2:** Correlation between study variables for male civilian sample.

Variable	QnC	QlC	IA	PT	E	ATWITM	A
Quantity contact	0.79						
Quality contact	0.28***	0.86					
Intergroup anxiety	−0.12**	−0.27***	0.83				
Perspective-taking	0.10*	0.31***	−0.19***	0.78			
Empathy	0.12**	0.35***	−0.15**	0.58***	0.89		
Attitudes towards women in the military	0.18***	0.35***	−0.19***	0.29***	0.24***	0.88	
Age	−0.09*	−0.04	−0.11*	0.04	0.04	−0.03	--
Mean	2.29	5.46	5.60	4.90	3.15	4.89	35.70
SD	0.70	0.89	1.10	0.78	0.50	1.14	11.77

QnC, quantity contact; QlC, quality contact; IA, intergroup anxiety; PT, perspective-taking; E, empathy; ATWITM, attitudes towards women in the military; A, age; YATC, years at the college. Cronbach’s alpha for measures on the diagonal.

*p < 0.05; **p < 0.01; ***p < 0.001.

Contact quantity was significantly associated with intergroup anxiety and empathy, but not perspective-taking. Intergroup anxiety and perspective-taking were significantly associated with attitudes toward women in the military, while empathy was not. The direct effect between contact quantity and attitudes toward women in the military was significant, while intergroup anxiety demonstrated a significant indirect effect.

Contact quality was significantly associated with intergroup anxiety, perspective-taking, and empathy. Intergroup anxiety and empathy were not significantly associated with attitudes toward women in the military, while perspective-taking was significantly related to attitudes toward women in the military. The direct effect between contact quality and attitudes toward women in the military was significant, while perspective-taking demonstrated a significant indirect effect.

Our findings reveal that for a male, civilian sample, both quantity and quality of contact with women in leadership roles are related to attitudes toward women in the military. This suggests that increasing the number of women in leadership roles so that greater interaction can occur as well as increasing the opportunity for greater quality interactions are important to changing attitudes toward women in the military. Given the few female leaders there are in various domains such as sport (Evans and Pfister, 2021), medicine (Stephens et al., 2020), and the military (Government of Canada, 2023), finding ways to increase representation across multiple leadership roles within society may have important consequences for attitudes toward women serving in nontraditional roles such as in the military.

Intergroup anxiety demonstrated significant indirect effects for quantity contact and attitudes toward women in the military. Perspective-taking demonstrated significant indirect effects for the quality contact-attitude relation. These findings suggest that different mechanisms for these two types of contact may be operating. Perhaps for men more exposure to women in leadership roles reduces perceived threat of those women while quality of interactions is important to understand and actively take on the perspective of others.

Empathy alone did not appear to play an important role in the relation between contact and attitudes toward women in the military. This may be because we incorporated a general measure of empathy. Including a specific measure that focusses on the target group (e.g., Aberson and Haag, 2007), may produce different findings, however that has not been empirically tested. Unfortunately, employing a measure of empathy that identifies the target outgroup may result in shared variance between the empathy measure and the dependent variable because the outgroup was identified in both measures. This is particularly problematic when respondents are taking the survey in one sitting.

## 4 Study 3

It is important to study attitudes of women toward women in the military in a civilian, female sample as women assist and support the overall culture and recognition of women’s roles and rights. Gender is a predictor of attitudes toward women in the military (Vogt et al., 2007), thus it was important to assess the models with each gender to determine if the models function in a similar fashion.

For women, we expected only quality of contact would predict attitudes toward women in the military. Quality contact appears to be more effective in improving attitudes in some research (e.g., Tam et al., 2006; Prestwich et al., 2008; Kanamori et al., 2022; Zhang et al., 2023). As the contact group is women in leadership, women share one important characteristic with this contact group, gender. Because of the shared gender, the strength of the association between contact quantity and attitudes toward women in the military may be reduced for the female sample (given the strength of quality contact-attitude relation in other studies, we do not expect the shared association of gender to reduce the quality contact-attitude association to nonsignificance here). Similarly, we do not expect intergroup anxiety to play a role for the female sample but we do expect perspective-taking and empathy to demonstrate significant indirect effects. Marsden and Barnett (2020) found that empathy and perspective-taking acted as mediators between social political ideology and sexual prejudice, with empathy functioning as a mediator for women only and perspective-taking functioning as a mediator for both women and men.

*H5:* For a civilian female sample, quality contact would predict attitudes toward women in the military. Perspective-taking and empathy would demonstrate significant indirect effects.

### 4.1 Method

#### 4.1.1 Participants and procedure

This study received approval from the RMC Research Ethics Board. The participants were recruited through Prolific. Only self-identified Canadian women were asked to voluntarily participate. All procedures employed were identical to study 2.

A total of 407 participants clicked on the survey study link but only 374 participants’ data were complete. Six consented to the survey but then did not answer any questions, 11 identified their gender as “other,” 1 who identified as a man, 1 who did not confirm their gender, 1 who indicated they had been in the military, 13 failed one or more attention check items. The participants’ ages ranged between 19 to 75 (*M* = 34.05, *SD* = 11.82, median of 31) with 0.3% not having a high school education, 16.8% having completed high school as their highest level of education, 15.5% having completed a certificate, 48.1% having a bachelor’s degree, and 19.3% with a post undergraduate education. The average number of years of work experience was 13.92 (SD = 10.58) with a median of 11 years, 92.8% declared English as their first language, 3.2% declared French, and 3.7% declared other. The range for contact quantity scores (total scores) was 5 to 23 in this sample, while contact quality (total scores) was 8 to 42.

#### 4.1.2 Measures

All measures were identical to those described in Study 2, except for the Stephan and Stephan’s (1985) intergroup anxiety scale. The instructions were modified to read: “If you were interacting with women (e.g., talking with them, working on a project with them), how would you feel compared to occasions when you are interacting with only men?”

### 4.2 Results and discussion

One-tailed Pearson correlations were calculated between the study variables (see Table 3). Correlations were based on 1,000 bootstrap samples. Significant intercorrelations were found for all study variables except empathy was not significantly related to contact quantity.

**Table 3:** Correlation between study variables for female civilian sample.

Variable	QnC	QlC	IA	PT	E	ATWITM	A
Quantity contact	0.76						
Quality contact	0.31***	0.90					
Intergroup anxiety	−0.23***	−0.43***	0.90				
Perspective-taking	0.15**	0.25***	−0.15**	0.77			
Empathy	0.08	0.20***	−0.15**	0.46***	0.88		
Attitudes towards women in the military	0.07	0.24***	−0.12*	0.10*	0.14**	0.74	
Age	−0.12**	−0.08	0.17***	−0.01	0.04	0.10*	--
Mean	2.41	5.48	4.69	5.03	5.53	5.47	34.05
SD	0.71	1.01	1.51	0.75	0.79	0.78	11.82

QnC, quantity contact; QlC, quality contact; IA, intergroup anxiety; PT, perspective-taking; E, empathy; ATWITM, attitudes towards women in the military; A, age; YATC, years at the college. Cronbach’s alpha for measures on the diagonal.

*p < 0.05; **p < 0.01; ***p < 0.001.

As in Study two, two indirect effects models were tested to determine whether intergroup anxiety, perspective-taking, and empathy accounted for unique variance in the association between quantity contact or contact quality and attitudes toward women in the military. These models were run with 95% confidence intervals and 5,000 sample bootstrapping.

**Figure 3:**
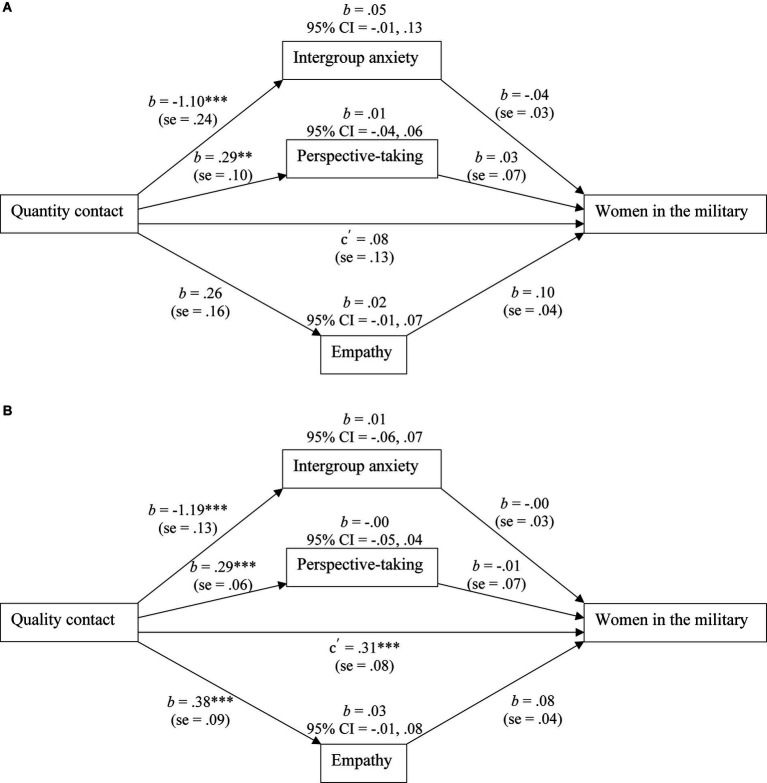
Women civilian sample: the indirect effect test of intergroup anxiety, perspective-taking, and empathy on the association between quantity contact **(A)** or quality contact **(B)** and attitudes towards women in the military. All path values represent unstandardized regression weights. CI = confidence interval. *p < 0.05; **p < 0.01; ***p < 0.001.

See Figure 3 for the findings of the first model. Contact quantity was significantly associated with intergroup anxiety and perspective-taking, but not empathy. Empathy was related to attitudes toward women in the military, however, intergroup anxiety and perspective-taking were not. The direct effect between contact quantity and attitudes toward women in the military was not significant; all indirect effects were also not significant. Thus, for women, as expected, quantity contact with women in positions of authority and intergroup anxiety did not demonstrate significant relations with attitudes toward women in the military.

Contact quality was significantly associated with intergroup anxiety, perspective-taking, and empathy, but none of the three explanatory variables were related with attitudes toward women in the military. The direct effect between contact quality and attitudes toward women in the military was significant (see Figure 3).

Unexpectedly, perspective-taking and empathy did not demonstrate any significant indirect effects between contact quality and attitudes toward women in the military. It is unclear why these two intermediary variables did not demonstrate significant indirect effects. Perhaps, because of the shared gender between participant and target outgroup member, these variables cannot explain the relation. Alternatively, perhaps different intermediary variables need to be studied in the future, such as trust (Hodson, 2011) or perceived effectiveness in leadership roles (Wood and Charbonneau, 2018). These findings suggest that, like men, women need to have quality interactions with women in positions of power to have favorable attitudes toward women working in the military.

## 5 General discussion

As organizations seek to integrate women into various positions that have been dominated by men, determining which factors improve attitudes toward women working in predominantly male domains, such as the military, is important. More specifically, identifying the factors that may increase acceptance of women working in the military can change recruitment practices and treatment of women in the military. We wished to determine whether quality and quantity contact with women in positions of power or authority reduces intergroup anxiety, increases empathy and perspective-taking, and subsequently creates more favorable attitudes toward women in the military. This was examined with a military sample consisting of men, a civilian sample of men, and a civilian sample of women. Our findings revealed that quantity contact was directly related to attitudes toward women in the military for the male civilian sample, and quality of contact was related to favorable attitudes toward women in the military across all three samples. For civilian men, intergroup anxiety demonstrated a significant indirect effect between quantity contact and attitudes toward women in the military, and perspective-taking demonstrated a significant indirect effect between quality contact and attitudes toward women in the military. Intergroup anxiety, perspective-taking, and empathy did not demonstrate any significant indirect effects for the sample of women.

It is important to examine attitudes regarding women in the armed forces, as the military has historically been considered a “masculine” profession (Munshi and Pandey, 2017; Deng et al., 2023a,b) and therefore it is considered atypical for women to serve in military roles. This is despite the fact that women are successful in military and leadership roles (Boldry et al., 2001; Eagly and Carli, 2003; Paustian-Underdahl et al., 2014; Chadwick and Dawson, 2018; Farh et al., 2020). Women can be perceived as the “outgroup” in professions primarily occupied by men. Because of this they may not be deemed suitable by individuals who have few experiences interacting with women in important and powerful roles. Military personnel, officers or noncommissioned members, exercise important leadership functions within the military (Morath et al., 2011) so leadership forms an important characteristic required of a military member. Thus, women can be perceived as outgroup members within the military based on two factors: their gender and taking on a career that requires leadership qualities.

Because women are not viewed as suitable for military positions, they may experience overt and indirect obstacles to being integrated within various nontraditional gender roles and when climbing rank in the military. The military is a masculine culture which serves as an immediate roadblock to promotion and empowerment for women (Ryan, 2008). This is seen in many military organizations and peacebuilding operations where women occupy traditional gender roles (Mazurana, 2003; Office of the Auditor General of Canada, 2016). Militaries have been designed by men primarily in response to potential threats of countries ruled by men (Ryan, 2008). Gender roles and the masculine culture of the military shape the level of acceptance for women occupying different roles within the military (Munshi and Pandey, 2017; Karazi-Presler et al., 2018; Deng et al., 2023a,b).

Women are considered not suitable for the military because of a variety of myths: they are not physically fit enough, there is a negative public view of female casualties, women influence the unit cohesion and morale in a negative manner, standards have to be lowered when permitting women in the military, men will have to cover the slack for women, and because gender integration is driven by politics rather than need or best practices (Munshi and Pandey, 2017). However, women play an important and positive role in the military and peacebuilding operations and have the potential to offer a different perspective if given the freedom to do so and given the freedom to help shape and change the military. Research has demonstrated that board gender diversity is related to performance when there is contact between men and women (Havrylyshyn et al., 2023). Increasing contact with women can increase openness and prosocial attitudes in patriarchal cultures with strong gender segregation (Jones, 2023). Mazurana (2003) noted that, In all of these operations, “women police peacekeepers were seen by locals as less threatening, more willing to listen, and better able to diffuse potentially violent situations.” (p. 67) Furthermore, “compared to their male colleagues, women police officers have significantly lower rates of complaints of misconduct, improper use of force, or inappropriate use of weapons.” (p. 65) Having women in the military is crucial as having more women in male-dominated disciplines can help signal to other women that they are welcome (Olsson and Martiny, 2018; Porter and Serra, 2020; Soules, 2020). It can also influence women’s performance (Thompson and Sekaquaptewa, 2002), and may help change the culture of these organizations as they provide alternate opinions, perspectives, and lived experiences.

Women are underrepresented in civilian and military leadership positions and do not occupy equal status with men in many domains (e.g., Lawson et al., 2022; Inter-Parliamentary Union, 2023). This creates a vicious cycle where women are believed to not be able to perform as well as men in those roles; fewer women then apply to be in those positions (Olsson and Martiny, 2018) and thus there are fewer women in leadership positions. One reason for women occupying fewer such roles is they do not see themselves in positions that have been constructed for and by men. Because there are few women in those roles, this propagates the view that either they are not effective in those roles, or those positions are primarily constructed for men (Eagly and Wood, 2012; Olsson and Martiny, 2018). Increasing quality contact with women in positions of authority can increase men and women’s acceptance of women in the military. Munshi and Pandey (2017) indicate that many male soldiers, who come from environments where there are few or no female leaders, have a difficult time accepting orders from female officers. This suggests that contact with women leaders is essential to accepting women in the military (Munshi and Pandey, 2017). Given the importance of quality of contact and the lack of women in leadership positions, women in leadership roles may have an added unintentional burden of acting as the spokesperson or role model for all women in those positions. Thus, it is important to maintain efforts at training and mentoring women for leadership roles. Creating more opportunities for women to occupy leadership roles and providing support to act as role models may result in greater quality contact with women in positions of authority.

More broadly speaking, favorable attitudes toward women in the military could have spillover effects in attitudes toward other outgroup members such as ethnic and sexual minority groups. Evidence exists that prejudice can be generalized; individuals who express negative attitudes toward sexual minorities, for instance, also express more negative attitudes toward other minority groups and to women (Bergh and Brandt, 2023). Furthermore, increasing favorable attitudes toward women in the military could lead to accepting attitudes and behaviors that are not based on strict hegemonic masculine roles within the military. This could provide greater flexibility for men to express themselves beyond the limited gender roles typically associated with the military.

Although not studied here, contact may be important for reducing sexual harassment which is particularly problematic in the military (Buchanan et al., 2014; Deschamps, 2015; Daphna-Tekoah et al., 2021; Arbour, 2022). Having negative attitudes toward women in atypical gender roles is related to having more favorable attitudes toward sexual harassment (e.g., Foulis and McCabe, 1997; Flood and Pease, 2009) and viewing sexual violence and intimidation as less coercive (e.g., Burgess and Borgida, 1997). High sexual harassment rates, and the use of harassment to keep women in subordinate positions (Van Wijk, 2011) may be reduced with greater contact experiences. For instance, Taschler and West (2017) found that contact with women in positions of power or authority resulted in less sexism and a reduction in rape myth acceptance. Thus, creating greater contact experiences may have the added benefit of improving the climate of these work environments.

### 5.1 Limitations and future directions

Conclusions from these findings need to be tempered by the limitations of the research. First, only Canadian samples were studied, and a small, young, male military sample was included, thus limiting the generalizability of the results. Common method variance could influence the findings as all instruments were taken by the participants in one sitting. Other intermediary variables and relations were not incorporated because of the limited sample sizes. For instance, some researchers found perspective-taking led to improved empathy which led to more favorable attitudes toward outgroup members (e.g., Hodson et al., 2009; Matera et al., 2021). While others found perspective-taking to mediate the relation between empathic concern and attitudes toward outgroup members (e.g., Miklikowska, 2018). Regardless, the findings support the importance of further examining the role of contact in changing attitudes toward women.

Future research should examine different ways to increase contact relationships with women in leadership positions. Research has shown that direct cross-group friendships and extended cross-group friendships (knowing that a friend has a friend belonging to a minority group, for instance) can reduce prejudice (Vonofakou et al., 2008). Research examining this with women in leadership positions should study direct and extended cross-group friendships. Perhaps creating virtual training experiences with women in leadership positions would improve attitudes as there is some evidence for the effectiveness of virtual contact (Tassinari et al., 2022). Actual contact situations can be difficult to implement, however, imagined contact experiences have been shown to be effective for improving attitudes to various outgroup members (Miles and Crisp, 2014). Specifically, imagining contact with leaders improved attitudes toward leaders (Meleady and Crisp, 2017). Thus, exposure to female leaders in different manners should be explored.

Even though people are in contact with women, our findings suggest they require different kinds of contact experiences in order to be able to fully accept the myriad of roles women occupy in society. For instance, women are underrepresented in STEM fields (Friedmann and Efrat-Treister, 2023); increasing contact experiences with women in STEM may change attitudes regarding women’s ability to work in those fields or even change attitudes regarding the necessary qualities to be successful in those fields. This suggests that it is important to assess the role of contact for other culture and minority group members in leadership positions. Certainly, ingroup members may develop more favorable attitudes toward outgroup members after experiencing contact with them. However, this may not translate to positive attitudes about the outgroup members’ ability to occupy positions of authority within different realms of society. This research suggests the importance of pursuing this line of research for women and minority groups in different professions.

Our studies suggest additional research is needed into contact theory and the importance of studying quality and quantity contact separately. For civilian men, both quality and quantity contact demonstrated significant direct effects. Thus, not only is the quality of that relation important, but the number of those relationships are also important. For civilian women and our military sample of men, only quality of contact was important. What could explain the differences found for quantity and quality contact? The shared similarity on one of the group characteristics, such as gender (for the civilian female sample), or serving a leadership role (for the male military sample), may influence the strength of the relationship between quantity contact and the dependent variable. If we are to categorize women in the military as an outgroup, then for civilian men, both gender and the position (leadership) form the outgroup categorization. For civilian women, only the leadership position may be considered part of the outgroup, while for military men, only gender may be the “outgroup” component. Thus, for quantity contact to contribute unique variance to the dependent variable, it perhaps requires more than one outgroup categorization in order to be effective, while this is not required for quality contact. Additional research should explore dual or multiple outgroup memberships and whether each outgroup membership is salient in the contact-prejudice relation.

Another reason for why quantity contact did not correlate with attitudes toward women in the military may be due to the military being a hierarchical organization (Nicol et al., 2007; Crowley and Sandhoff, 2017; Taber, 2018; Pendlebury, 2020; Richard and Molloy, 2020; Davis, 2022; Deng et al., 2023b). Kende et al. (2018) found that the contact-prejudice relation was reduced in cultures that endorse hierarchy. Thus, it is possible that the quantity contact-prejudice relation in the military sample was attenuated as the members embrace social dominance and hierarchy. This would need to be studied further.

Our findings suggest the intermediary variables’ roles differ depending upon the sample. For instance, whether intergroup anxiety or perspective-taking demonstrated significant indirect effects depended on the sample and whether contact quantity or quality were the predictors. For civilian women, intergroup anxiety, perspective-taking, and empathy did not demonstrate significant indirect effects in our models. Thus, quality contact may operate via different mediators not studied here. One important mediator may be hostile or benevolent sexism (Glick and Fiske, 2001) as both predict attitudes toward women in the military (Young and Nauta, 2013) and support for a masculine military structure (Sakallı Uğurlu and Özdemir, 2017). For civilian men, the more exposure men have with women in positions of authority may help reduce anxiety over their perceived threat. Perspective-taking demonstrated a significant indirect effect between quality contact and attitudes toward women in the military for civilian men. This is perhaps because quality contact provided them with a richer understanding of women in leadership roles and therefore they view women as acceptable in these positions. Empathy played no role for any of the models except in its correlation with the predictor or dependent variable. This suggests it is important to study perspective-taking and empathy separately. Combining them may reduce the strength of the indirect effect of perspective-taking and mask any potentially important individual effects.

Research should explore alternate reasons for why women do not join the military. Our work suggests there may be a relation between access to women in leadership positions and attitudes toward joining the military. Other research could examine whether women view the military as a male-dominated context where they do not fit in. Perhaps perceptions of self-efficacy in a military environment play a role (Wood and Charbonneau, 2018). Finally, research has shown that women leave the military due to familial obligations (King et al., 2020); expectations of having greater familial responsibilities may impact their decision to join.

## 6 Conclusion

Despite the fact that the majority of military occupations were opened to women in Canada in 1989 and women could participate in combat roles since the 1990s (Statistics Canada, 2022), the number of women in the military has not markedly increased in the past decade (Office of the Auditor General of Canada, 2016). When women do join, their occupations are in stereotypical roles (Office of the Auditor General of Canada, 2016). Similarly, the number of women in leadership positions outside the military are limited (e.g., Lawson et al., 2022; Inter-Parliamentary Union, 2023). These low numbers limit the number of interactions the public and military members can engage in and limit the perceived ability of women to successfully operate in a wide array of positions. A limited number of opportunities to engage with or view women in leadership positions creates a situation whereby women do not apply to those positions as they do not see themselves in those roles or capable of working in them and men see themselves as more effective than women (e.g., Paustian-Underdahl et al., 2014; Lawson et al., 2022). Engaging more women in those roles and providing opportunities for those women to interact with others can improve attitudes toward women in leadership or male-dominated positions. This may change the nature of those positions and organizations as both women and men gain a more expansive appreciation of the various ways in which leadership can be enacted. The military needs to change (Deschamps, 2015; Government of Canada, 2021; Arbour, 2022), as it can no longer pander to a hegemonic masculine view of how it should operate (Deng et al., 2023b). Increasing the quality of interactions is one way to enact those changes.

## Data availability statement

The datasets presented in this study can be found in the online OSF repository. Access can be obtained through https://osf.io/z9juc.

## Ethics statement

The studies involving humans were approved by Royal Military College of Canada Research Ethics Board. The studies were conducted in accordance with the local legislation and institutional requirements. Written informed consent for participation was not required from the participants or the participants’ legal guardians/next of kin because participants were first informed of the study in writing on Survey Monkey, and all participants were over 18 years of age. Should they agree to participate in the study, they needed to indicate yes on the form and continue with the study. Thus, they were informed that by proceeding with the study they had given consent. A formal letter was not signed in order to maintain anonymity of all participants.

## Author contributions

AdN: Conceptualization, Formal analysis, Funding acquisition, Methodology, Project administration, Resources, Supervision, Writing – original draft, Writing – review & editing. AmMN: Data curation, Visualization, Writing – review & editing.
